# Arboreal or terrestrial: Oviposition site of *Zhangixalus* frogs affects the thermal function of foam nests

**DOI:** 10.1002/ece3.10926

**Published:** 2024-03-05

**Authors:** Yukio Ichioka, Hisashi Kajimura

**Affiliations:** ^1^ Laboratory of Forest Protection, Graduate School of Bioagricultural Sciences Nagoya University Nagoya Japan

**Keywords:** amphibian, egg survival, foam nest, Japanese green tree frog, oviposition site, reproductive plasticity, temperature, *Zhangixalus arboreus*

## Abstract

Temperature is essential for the survival and development of eggs. Some anurans have evolved and developed foam nesting traits, with thermal insulation considered to be among their functions. Foam‐nesting frogs tend to exhibit reproductive plasticity. For example, they oviposit on both trees and the ground. How such plasticity affects foam nest function is of major relevance and is likely related to the adaptation of foam nesting frogs. However, this has not been well studied. In this study, we examined the interaction between foam nest site, foam nest function, and egg fate using the Japanese green tree frog, *Zhangixalus arboreus*, and analysed how nest site differences (arboreal or terrestrial) affect the thermal function of foam nests. We compared the thermal functions of foam nests between arboreal and terrestrial oviposition sites of *Z. arboreus*. We artificially replaced half of the arboreal nests with terrestrial environments and recorded temperature in and outside of the experimental terrestrial nest and original arboreal nests. We also examined egg survival and hatching rates for all the nests. The results indicated superior heat insulation in terrestrial nests, with warmer temperatures inside than outside the nests, especially at night, which led to a high egg survival rate. Therefore, terrestrial ovipositing should be valid under cold weather conditions. This may be related to the evolutionary history of oviposition site plasticity of this genus, which originally had an arboreal oviposition trait but evolved into terrestrial site use owing to global cooling. Our novel insights into the evolution and adaptivity of foam nesting and oviposition site use in *Z. arboreus* make an important contribution to animal ecology.

## Graphical abstract

Some foam nesting anuran has oviposition site plasticity. We revealed that arboreal or terrestrial site use affects the thermal function of the foam nests in *Zhangixalus arboreus*. Such a site depending on thermal function may relate to the evolution of oviposition site use.

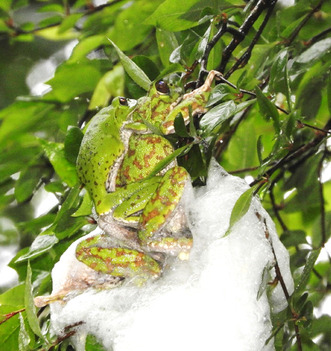

## 1 INTRODUCTION

Among oviparous animals, egg development speed and survival are influenced by external temperature (Bernal & Lynch, 2013; Carroll, 1996; Licht, 1971; Scheffers et al., 2013). Therefore, animals should attempt to provide a better thermal environment for their eggs to achieve high fitness. Oviposition site selection is a strategy used to maximise the chance of offspring experiencing optimal thermal conditions (Pruett et al., 2020). Some animals have also evolved traits that influence the thermal conditions of their eggs.

Anurans are known for their diversity of reproductive modes (Haddad & Prado, 2005; Nunes‐de‐Almeida et al., 2021). A particular mode is froth nesting, which is observed across several families, including Hylidae (Haddad et al., 1990), Rhacophoridae (Matsui & Maeda, 2018), and Leptodactylidae (Hödl, 1992; Méndez‐Narváez et al., 2015). Froth nests are made of adult secretions. Secretions are aerated to form a spawn body that encloses eggs throughout some or all stages of egg/(and even tadpole) development. The nests have various functions, such as defence against predation, prevention of egg desiccation, oxygen supply and acting as potential food for larvae (Gould et al., 2019, 2021; Kusano et al., 2006; Seymour & Loveridge, 1994; Tanaka & Nishihira, 1987; Zina, 2006). Froth nesting can also provide thermal benefits for eggs by insulating them from external fluctuations in temperature, and keeping them either warm or cool (Dobkin & Gettinger, 1985; Downie, 1988; Kusano et al., 2006; Philibosian et al., 1974; Rodrigues et al., 2020; Shepard & Caldwell, 2005). However, few studies have focused on nest construction site effects on these functions.

Froth nests are classified into two types, foam nest and bubble nest (Altig & McDiarmid, 2007). Some foam‐nesting anurans exhibit reproductive mode plasticity, utilising multiple environments, such as trees and the ground, for oviposition (Fukutani et al., 2020; Matsui & Maeda, 2018). Site‐use plasticity may affect the functional properties of the foam nests. For example, Méndez‐Narváez et al. (2015) reported site‐dependent thermal function differences between nests on the water surface and land in *Physalaemus fischeri*. However, no studies have addressed the matter with regard to other families or site‐use combinations (e.g. arboreal and terrestrial). This means there is still limited understanding of how intraspecific oviposition site plasticity affects foam nest function and egg fate among anurans.

**FIGURE 1:**
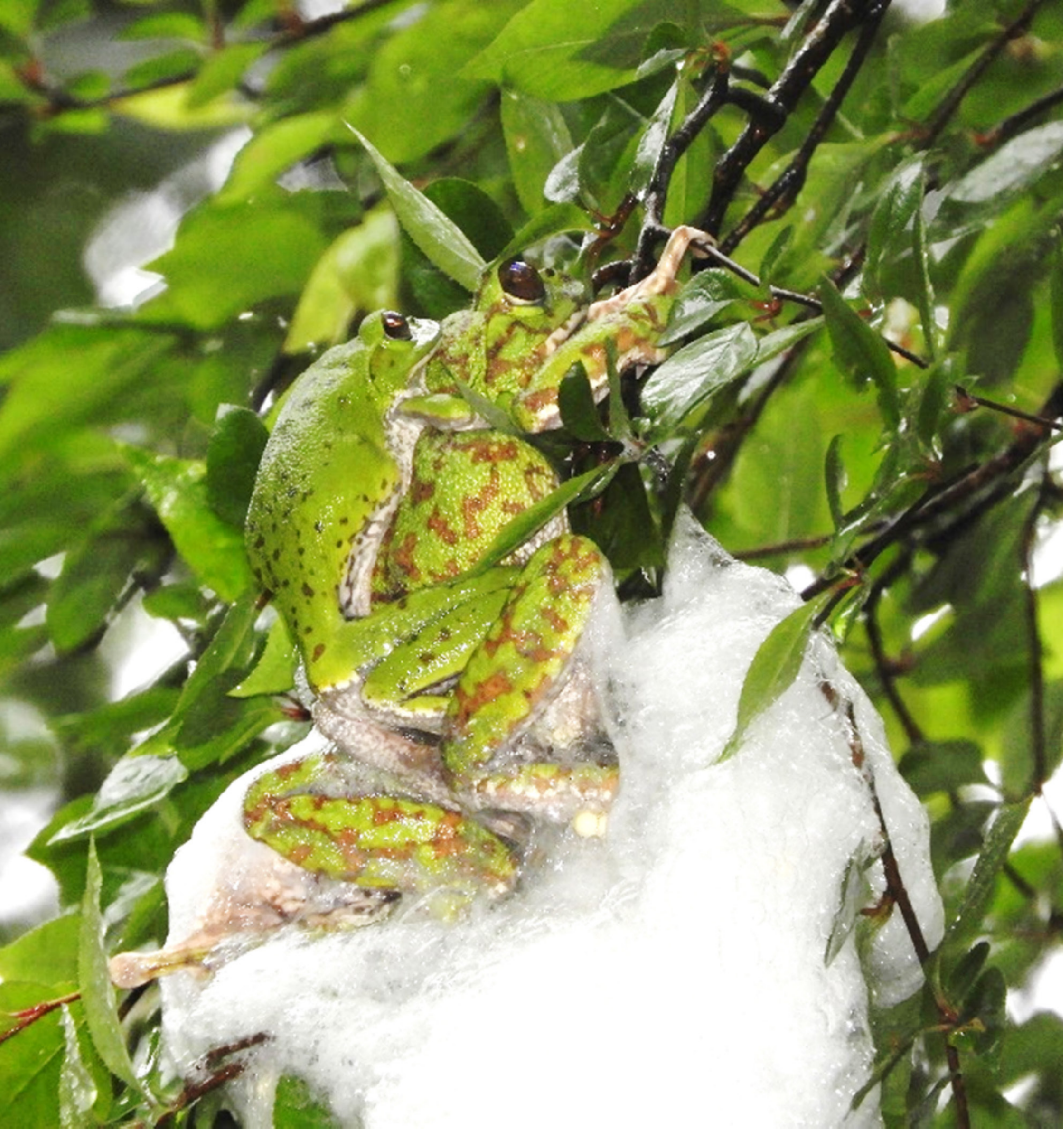
Reproductive behaviour of *Zhangixalus arboreus* above the pond in Nagoya University Forest (Inabu field), central Japan. The female (right side) moved her hind legs and made the foam nest on a *Pourthiaea villosa* var. *leavis* tree.

Herein, we studied the effect of foam nest site, foam nest function and egg fate using the Japanese green tree frog, *Zhangixalus arboreus*. This is a relatively large frog endemic to Japan that shows plasticity in nesting site use that includes both trees and the ground (Figure 1; Ichioka & Hijii, 2021; Matsui & Maeda, 2018). Among *Zhangixalus* species, arboreal nest‐making is an ancestral behaviour, with terrestrial nest‐making having evolved during subsequent periods of global cooling (Dufresnes et al., 2022). Therefore, we anticipated that terrestrial foam nesting may provide certain benefits in cooler environments, such as higher heat retention, compared to arboreal foam nesting. We verified this hypothesis by artificially imitating arboreal and terrestrial oviposition site. Additionally, we compare egg fate between arboreal and terrestrial sites.

## 2 MATERIALS AND METHODS

### 2.1 Study site

This study was conducted in one ephemeral pond at the Forest of Nagoya University (Inabu field), central Japan (35°11′ N, 137°33′ E; 980–1000 m asl). The pond was often dry during this survey period but reached a maximum size of approximately 16 m × 2.5 m (also contains a branch approximately 1 m × 4 m). The water depth at that time was 16 cm. In this region, the annual average temperature is 9.4°C, and the average annual precipitation is 2100 mm. This forest has been used as a conifer forestry area but has partly transitioned to natural vegetation including deciduous broadleaf trees.

### 2.2 Experimental design

**FIGURE 2:**
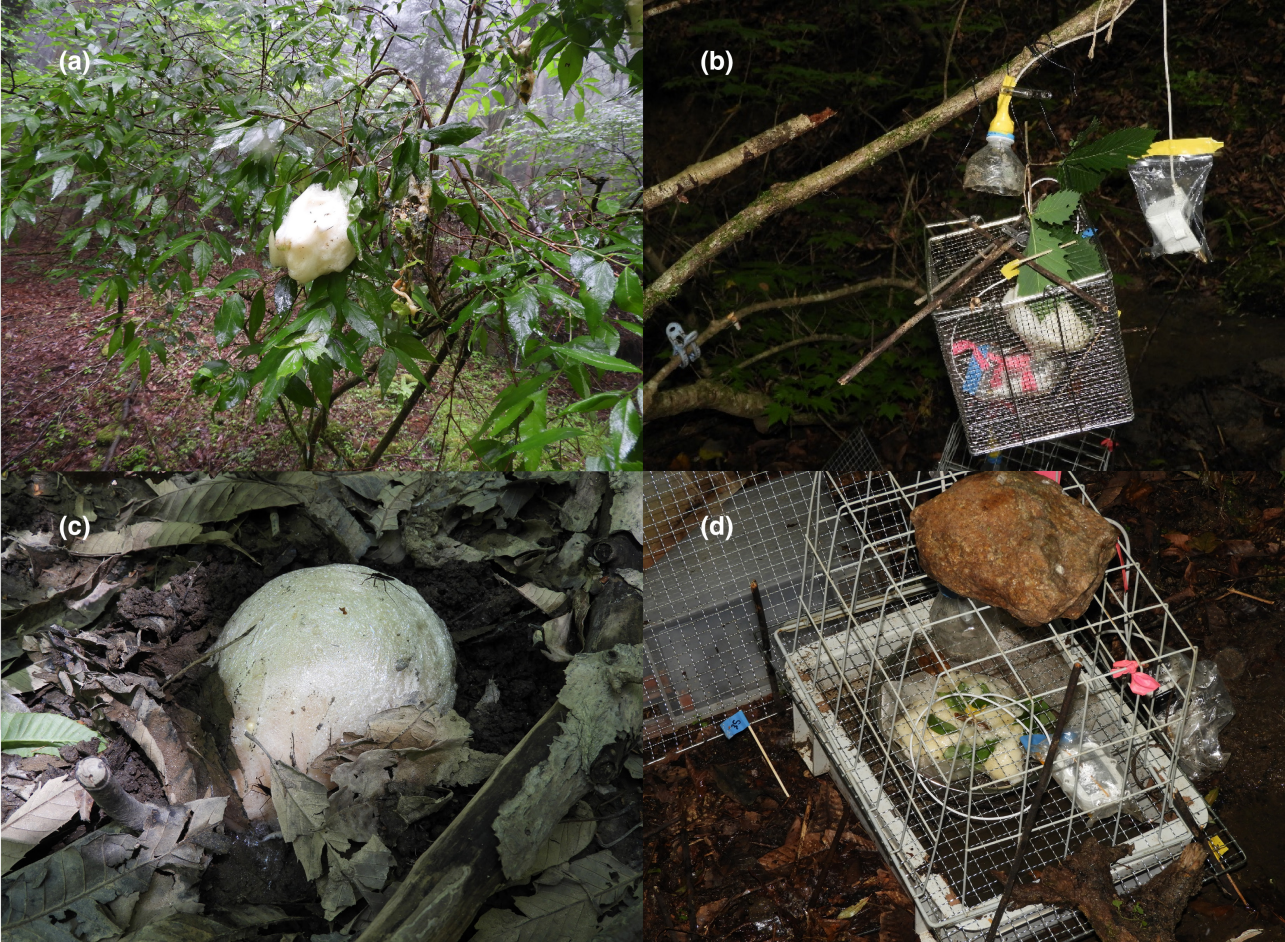
Natural and experimental foam nests of *Zhangixalus arboreus*. A naturally oviposited arboreal foam nest (a), our experimental setting of the arboreal nest (b), A naturally oviposited terrestrial foam nest (c) and our experimental setting of the terrestrial nest (d).

During the *Z. arboreus* reproductive season in 2022 (typically May to July), we collected fresh foam nests from a single pond within the study site. Searches for nests were conducted for 36 days from 18 May to 15 July, repeating the 3 consecutive days with 4‐day intervals. During the reproductive peak, we searched for foam nests for 14 consecutive days (13th–26th June). All nests were numbered using tags, and 10 fresh nests were used in further experiments after being transferred to arboreal or terrestrial sites (see Section 2.3), which imitated natural oviposition sites (Table 1). Only fresh nests were collected, which were identified based on their appearance, specifically the presence or absence of a white surface colour, that gradually changes to yellowish (Herpetological Society of Japan, 2021). In the study area, *Z. arboreus* use both arboreal and terrestrial oviposition sites (Figure 2a,c: Ichioka & Hijii, 2021). However, for further experiments, we only used nests spawned arboreally. This was to avoid any effect caused by the pre‐experimental site such as thermal condition. In our locality, amphibians such as frogs are not included in the list of experimental animals, which require approval for use in experiments. Furthermore, we did not injure any eggs or tadpoles, nor did we restrict the swimming actions of tadpoles.

**TABLE 1:** Basal information of every foam nest used in the experiment (placement site, discovery date, hatching day, egg number, hatching rate and period used for temperature measurement).

Nest number	Placement	Discovery	Hatching	Egg number	Hatching rate (%)	Recording period
Nest 6	Arboreal	27 May	20 June	398	21.1	6/1 to 3, 15 to 16
Nest 12	Arboreal	3 June	–	–	–	6/8 to 9
Nest 21	Arboreal	10 June	25 June	527	86.9	6/13 to 15, 16 to 21
Nest 23^a^	Terrestrial	13 June	26 June	377	94.4	6/14 to 19, 21 to 23
Nest 28	Terrestrial	19 June	29 June	322	99.1	6/19 to 23
Nest 30	Terrestrial	23 June	6 July	361	85.6	6/23 to 24, 26 to 29
Nest 31	Terrestrial	23 June	6 July	403	98.8	6/24 to 26, 6/29 to 7/1
Nest 32	Arboreal	23 June	6 July	421	87.2	6/23 to 7/1
Nest 34^a^	Arboreal	6 July	15 July	455	76.3	7/1 to 7/8
Nest 35^a^	Terrestrial	6 July	14 July	333	97.3	7/1 to 7/8

^a^ The construction day could not be confirmed for those nests. There may be a deviation of up to 4 days from the actual oviposition date.

### 2.3 Setting of nests

#### 2.3.1 Arboreal nests

From 27 May to 6 July, five foam nests were collected as arboreal nests on the day of discovery (Table 1). We cut off branches that foam nests belonged to, therefore each nest itself was not disturbed. Immediately, we tied the branch part of them to tree branches and hung on 1 m above the ground around the pond (Figure 2b). We used a dead tree standing nearby the pond as a hanging tree for one arboreal nest (nest 6), and other nests were hung on the acer tree (almost 3 m from the pond shore). This height is relatively low but is within the range of arboreal oviposition sites for *Z. arboreus* at this site (personal observation). These experimental nests were referred to as ‘arboreal nests’. The arboreal foam nest of *Z. arboreus* are typically deposited above the water surface, and the hatchlings drop from the nest into water (Matsui & Maeda, 2018). To catch those hatchlings and keep them alive, we set a bucket under each nest and poured water from a nearby river. Those buckets were 33 cm in diameter and 32 cm in height. We prevented overflow by making side holes at a height of 23 cm. As one experimental nest (nest 12) had been predated by raccoons from 15 June, we covered each nest with a wire mesh cage (cube of 200 mm each, 5.5 mm in stitch) to prevent animal predation, except for one nest (nest 6).

#### 2.3.2 Terrestrial nests

We imitated the pond edge environment that includes land and water in the plastic cases (37.5 cm × 28 cm × 15 cm) by adding soil (mainly consisting of humus from around the pond) and water, as this is the typical terrestrial oviposition location for *Z. arboreus* in the study area. From 3 June to 6 July, five foam nests that had been spawned on trees were collected as we did on arboreal nests and were transferred to plastic cases laid on the ground near the edge of the pond within the study site at the day of discovery (Table 1). One case was assigned to one nest and all nests were set on land part in each case. These experimental nests were referred to as ‘terrestrial nests’ (Figure 2d). Nylon mesh was spread under each nest to keep the hatchlings inside. Therefore, hatchlings could not flow into water as in the case of the natural terrestrial foam nest of the species (Matsui & Maeda, 2018). However, this enclosing did not hurt individuals because they could stay alive for a few days inside the melty foam, even if they were prevented to flow into the water. In addition, each nest was covered with wire mesh to prevent animal predation.

In this experiment, we introduced foam nests into controlled environments to avoid the complex effects of natural arboreal and terrestrial oviposition sites, which may influence the results. However, all nests were placed around a natural pond, as such locations are generally preferred for natural reproduction by adult frogs. Thus, we can assume that the different features between arboreal and terrestrial foam nests observed in this experiment are in line with those experienced in natural conditions.

#### 2.3.3 Temperature measurement

We inserted the thermal sensor of a data logger (2.2 mm in diameter, TR‐51i, T&D Corporation, Nagano, Japan) approximately 5 cm into each nest to record the internal nest temperature every 15 min. This depth was devised based on the general size of nests (88 mm in width and 120 mm in height: Matsui & Maeda, 2018), and was standardised to maintain a constant distance from the outside air. Simultaneously, we set another data logger (RTR‐503, T&D Corporation, Nagano, Japan) 10 cm above each nests to record the surrounding nest temperature every 15 min. As the number of experimental foam nests increased, we ran out of these loggers. Therefore, we recorded data from more nests by moving the instruments back and forth as needed. As a result, recordings were continued for 1–9 days at a time (Table 1).

We excluded all data taken within 30 min after all manipulations, such as data collection or replacement of the data logger, from further analysis because these may have some effects on the recording. In this experiment, the foam nests of *Z. arboreus* collapse some days after the hatching. Animal predation also causes a collapse of foam nests. Accurate measurement of the inside and surrounding nest temperature was not possible when the sensor was moved owing to nest collapse. Therefore, all data collected during the nest collapse were excluded from further analysis.

We calculated the difference in temperature (DT) between the inside of the nest and the surrounding air by subtracting the latter from the former. This score was used as an indicator of the thermal insulation effect of the nest. If DT > 0°C, the inside of the foam nest was kept warmer than the outside, and if DT < 0°C, the inside was kept cooler than the outside. During this process, we recorded all nest sizes (maxim width and depth) at the timing of setting using a digital calliper.

### 2.4 Egg survival and hatching rate per nest

For all arboreal nests and terrestrial nests, hatching success was monitored at the time when hatchlings began to swim inside the nests, causing them to collapse. The timing seems to coincide with that of natural foam nest collapse when hatchlings move onto the water environment. From collapsed nests or the backet under it, we got hatchlings and hatching success per nest was determined by taking photos and counting the number of hatchlings using ImageJ 1.41 (Schneider et al., 2012). Some unhatched eggs were observed in these photos and were counted. After taking photos, all hatchlings were immediately released into the ponds. The unhatched eggs and dead larvae remaining inside the nests were counted following dissection in our laboratory. Hatchlings were considered successfully hatched eggs, and all unhatched eggs and dead larvae were considered as failed eggs. The hatching success rate per nest was calculated for each nest based on the total number of hatchlings recorded compared to the total number of hatchlings and failed eggs.

### 2.5 Statistical analysis

R version 3.6.1 (R Core Team, 2019) was used for all analyses. The relationship between internal and external temperatures of nests located arboreally and terrestrially was analysed using Spearman's rank sum test. We compared the external temperature of nests between arboreal and terrestrial nests. Next, generalised linear model (GLM) analysis was conducted to determine how spawning sites affect the thermal conditions of foam nests. In this analysis, the temperature inside foam nests was the response variable (Family = Gamma), while the nest site (arboreal or terrestrial) and the temperature surrounding the foam nests were predictor variables.

The comprehensive egg hatching rates between arboreal and terrestrial sites were compared using generalised linear mixed model (GLMM) analysis. The survival of the egg was set as the response variable (family = binomial: live = 1, dead =0), and the nest site was set as the predictor variable. The random effect was the number of nests to which each egg belonged. In addition, the hatching rate per nest was compared between arboreal and terrestrial foam nests using the Wilcoxon rank sum test. For all analyses, the significance level was *p* < .05.

## 3 RESULTS

### 3.1 Temperature measurement

Thermal data was recorded at 1,929 times for arboreal nests and 1,964 times for terrestrial nests (Table 1).

The average, highest, and lowest temperatures of the arboreal nests were 16.8, 23.7 and 5.6°C, respectively (Table 2). The average, highest and lowest temperatures of the terrestrial nests were 17.9, 27.6 and 11.2°C, respectively (Table 2).

**TABLE 2:** Mean, maximum and minimum temperatures that were recorded inside and around the experimental nests.

	Arboreal nest		Terrestrial nest	
	Inside	Surrounding	Inside	Surrounding
Mean temperature (°C)	16.8	17.3	18.0	17.9
Maxim temperature (°C)	23.7	28.5	24.4	27.6
Minimum temperature (°C)	5.6	5.6	11.9	11.2

**FIGURE 3:**
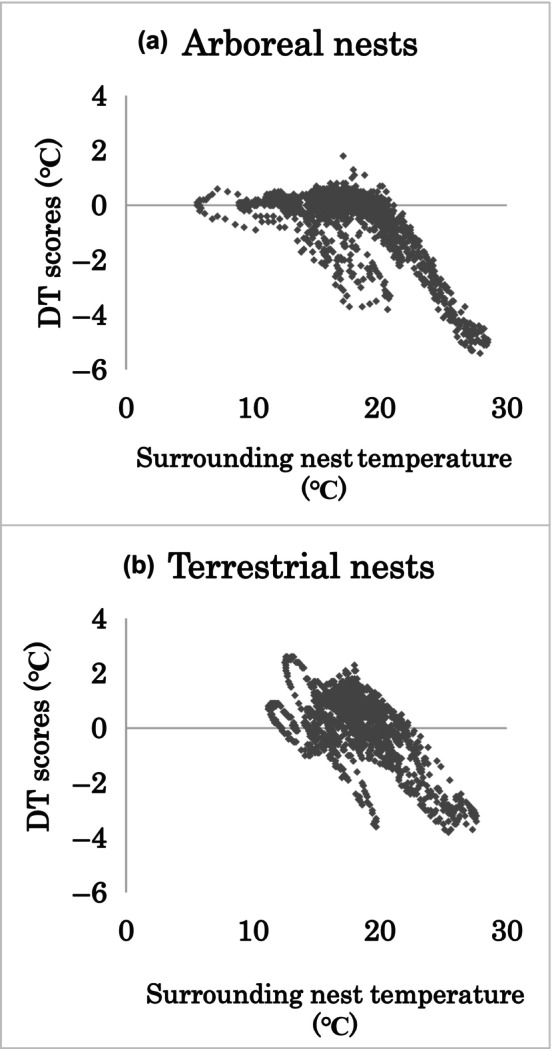
Relationship between difference in temperature (DT) scores and nest surrounding air temperature recorded at (a) arboreal nests and (b) terrestrial nests. DT scores were calculated by subtracting the surrounding nest temperature from the temperature inside nests at each recording (every 15 min).

**FIGURE 4:**
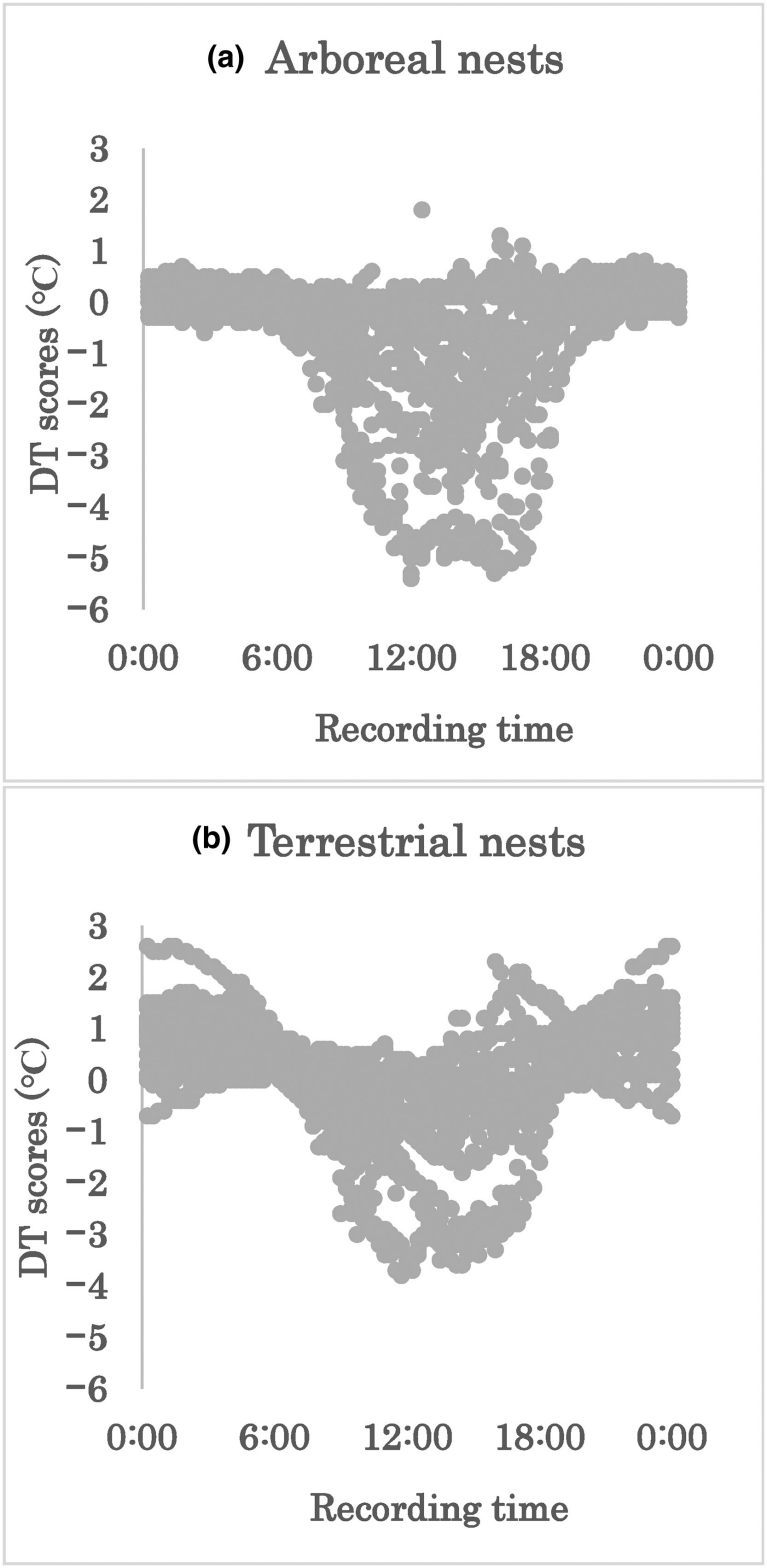
DT scores of (a) arboreal nests and (b) terrestrial nests at each recording.

**FIGURE 5:**
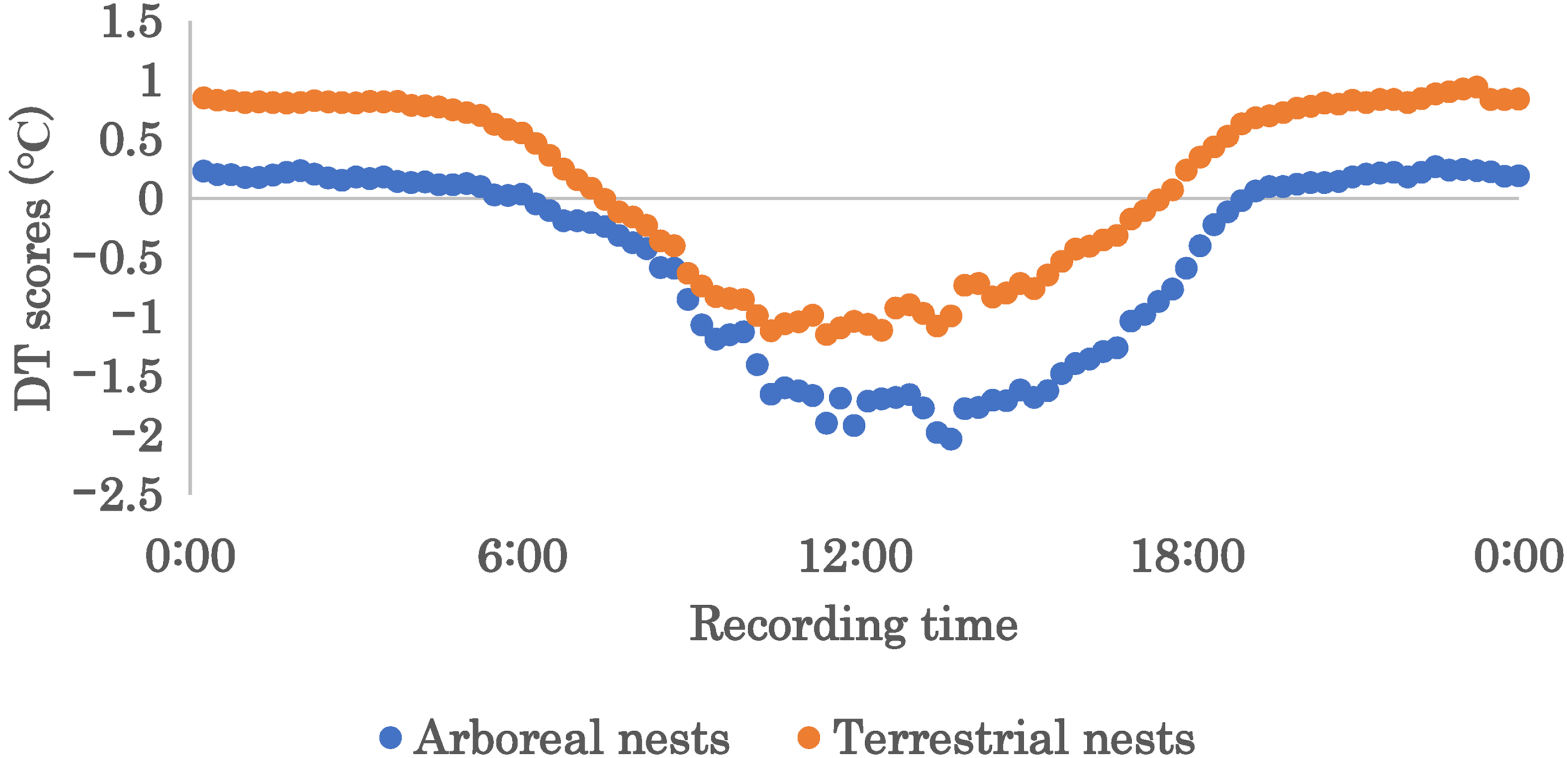
Average DT scores at each recording. Blue plots indicate those of arboreal nests, and orange plots indicate those of terrestrial nests.

The DT scores ranged from −5.4 to 1.8°C among the arboreal foam nests, with an average of −0.49°C. For the terrestrial foam nests, DT scores ranged from −3.8 to 2.6°C, averaging 0.13°C. A higher surrounding temperature contributed to a lower DT score in both arboreal and terrestrial foam nests (Spearman's rank sum test, *p* < .05, arboreal nests *R* = .53, terrestrial nests *R* = .36; Figure 3). In comparison, the surrounding temperature of nests was higher at terrestrial nests (Wilcoxon rank sum test, *p* < .05). The inside of experimental foam nests from both location was cooler than the surrounding temperature during the day, but was warmer at night (Figures 4 and 5).

GLM analysis revealed that both the nest site and surrounding temperature affected the inside temperature of experimental nests (Table 3). A high surrounding temperature contributed to a high internal temperature (*t* = 237.2, *p* < .001, Table 3). In addition, the nest site contributed to a higher inside temperature in terrestrial foam nests than in arboreal foam nests (*t* = 26.20, *p* < .001, Table 3).

**TABLE 3:** Results of generalised linear model (GLM) analysis revealing how the surrounding temperature of nests and nest placement site (arboreal or terrestrial) affect the temperature inside nests.

	Estimate	Std. error	*t*‐value	*p*‐value
Intercept	1.7278	0.05969	28.95	<.001
Surrounding temperature	0.8732	0.00367	237.2	<.001
Placement (terrestrial)	0.7140	0.02725	26.20	<.001

*Note*: Calculated *p*‐values indicate that both predictor variables had a significant effect.

Maximum width of nests were 112.4 ± 14.8 mm in arboreal nests, while 128.0 ± 10.2 mm in terrestrial nests (mean ± SD). Nest depth was 141.0 ± 42.6 mm in arboreal nests, while 91.4 ± 12.2 mm in terrestrial nests (mean ± SD).

### 3.2 Egg survival and hatching rate per nest

**FIGURE 6:**
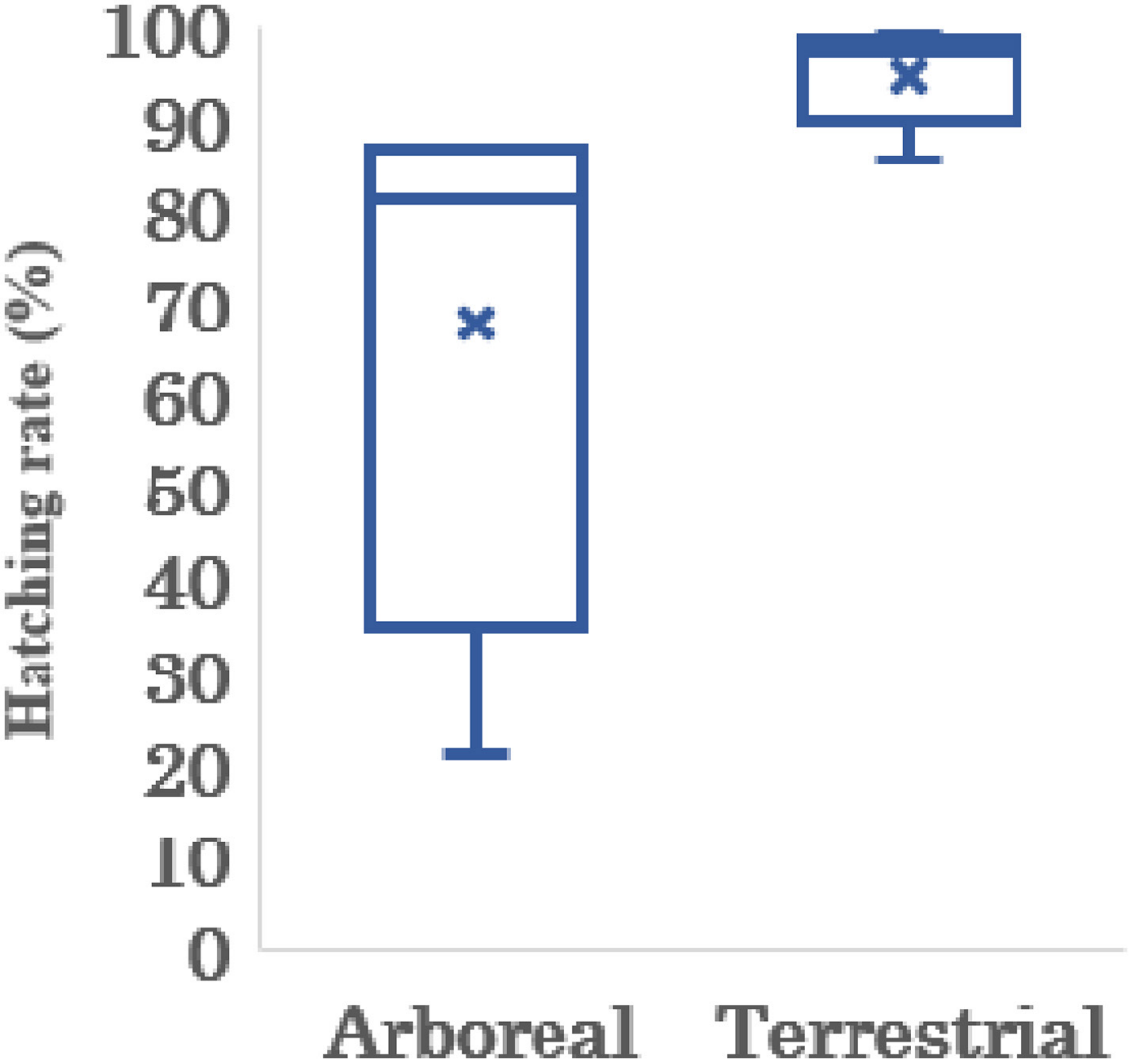
Comparison of hatching rate per nest between arboreal (*n* = 4) and terrestrial nests (*n* = 5).

Hatching rates per nest were 21.1%–87.1% (67.9% on average [*n* = 4]) for arboreal foam nests (Figure 6, Table 1). For terrestrial foam nests, the hatching rates per nest were 85.7%–99.1% (95.0% on average [*n* = 5]) (Figure 6, Table 1). In total, the arboreal nests contained 1,801 eggs, of which 1,256 successfully hatched. In terrestrial nests, 1,796 eggs were present, of which 1,706 successfully hatched. Nest 6, which was an arboreal foam nest, had the lowest hatching rate. In the nest, many maggots were observed inside the nest at the time of dissection. In addition, nest 12 crashed due to an animal attack (possibly a raccoon), and we were not able to calculate the hatching rate. As per GLMM analysis, the nest site significantly affected egg survival (*Z*‐value = 2.818, *p* < .05). Therefore, in terms of individual eggs, a higher survival rate was observed in terrestrial nests. This trend was similar even when nest 6, with very low hatching rates, were excluded from the analysis. In contrast, in the hatching rate per nest, there was no significant difference between arboreal and terrestrial foam nests (Wilcoxon rank sum test, *p* > .05).

## 4 DISCUSSION

*Zhangixalus* frogs have evolved oviposition site plasticity, with the capacity to deposit nests both arboreally and terrestrially. How this plasticity affects the function of foam nests or offspring fitness is rarely considered among anurans. We observed differences in the thermal insulation functions of foam nests as well as hatching rates. We believe that parental site selection could affect the thermal conditions that embryos experience.

We anticipated that terrestrial foam nesting would have benefits for developing eggs in cool environments, as previous studies have suggested that terrestrial oviposition evolved owing to global cooling (Dufresnes et al., 2022). We found that the temperature inside nests tended to be higher in terrestrial than in arboreal nests. Both the nest site and external temperature significantly affected the nest internal temperature. Furthermore, DT scores larger than 0°C were frequently observed in terrestrial nests compared to arboreal nests, suggesting that nesting location influences the temperature offspring experience over development.

As reported in a previous study, the inside of both arboreal and terrestrial nests tend to be lower than the outside under the high ambient temperature (Kusano et al., 2006). On the other hand, when the ambient temperature was low, the inside of terrestrial nests tended to be warmer than the surrounding air. This may have resulted in higher DT scores at night. On average, the inside temperature was nearly 1°C higher. In contrast, high DT scores were rarely observed in arboreal nests at any time. These site‐specific thermal features may have resulted from the surrounding environment. For example, terrestrial nests may have been blocked from the cold wind to some extent by the walls of the containers. This may have influenced the experimental results. However, even if there is no wall, it is quite possible that the wind will slow down due to surface vegetation. Therefore, we don't think there is a dramatic difference in wind speed between terrestrial oviposition sites in natural conditions and this experimental design. We also considered geothermal energy as a probable reason for this difference, since certain vertebrates utilise it in their nesting (Deméré et al., 2002; Tanaka et al., 2018). Heat from the ground may have been transferred to the terrestrial nests.

A previous study of *Z. arboreus* reported that low temperatures (13°C) result in decreased hatching rates (Kusano et al., 2006). Therefore, terrestrial nesting that will provide a warmer environment for the eggs is important in cold environments. This was especially relevant in our study, as the study site was a relatively cold mountainous area where the temperature typically drops to 10°C or lower.

Multiple studies have also reported that foam nests keep the inside of the mass cooler, and thus the eggs laid within (Downie, 1988; Gorzula, 1977; Rodrigues et al., 2020). The DT scores in our study decreased proportionately with the surrounding temperature. Therefore, it can be assumed that when the ambient temperature was high, both arboreal and terrestrial nests kept the inside cool. This was also observed in a previous study of arboreal foam nests in *Z. arboreus* (Kusano et al., 2006). This cooling function would be advantageous for egg survival on warm days because high temperatures are related to high egg mortality in amphibians (Carroll, 1996; Kuramoto, 1978; Kusano et al., 2006; Licht, 1971; Scheffers et al., 2013). In our study, the lowest DT score was recorded in arboreal nests, which suggests that the selection of arboreal sites contributes to a higher cooling function. However, further analysis is required.

In the GLMM analysis, a clear difference in egg survival was observed between arboreal and terrestrial nests, whereby terrestrial nests exhibited a higher egg survival. We believe that this could be due to the potential predators (maggots) (Gascon, 1991; Karraker, 2013; Kusano et al., 2005; Villa et al., 1982; Vonesh, 2000; Yorke, 1983) in arboreal nest 6, which had the lowest hatching rate per nest. However, even after excluding the egg survival data of nest 6, the nest site significantly affected egg hatching. These results suggest that terrestrial nests possess other features that contribute to improved egg survival. We believe that the warming of terrestrial nests positively affected the eggs inside, as study have found that low temperatures reduce the egg‐hatching rate of amphibians (Licht, 1971). Thus, the warming effect in terrestrial nests may have protected eggs from this risk. In addition, the contact with moist soil could have eliminated the risk of desiccation (Shangpliang et al., 2020), which has been found to be the main factor affecting egg mortality in foam nesting frogs (Allingham, 2017; Kusano et al., 2005).

Our research suggests that terrestrial foam nesting could contribute to protecting eggs from cold temperatures. This advantage seems to be related to the behavioural evolution of the genus *Zhangixalus*, which began nesting in terrestrial sites in response to global cooling (Dufresnes et al., 2022), despite its higher predation risk compared to arboreal nesting (Ichioka & Hijii, 2021). However, to fully elucidate the effect of the nesting site, additional aspects need to be considered. For example, predators are known to affect oviposition site selection (Giaretta & Facure, 2006; Magnusson & Hero, 1991), and various predators have been found to prey on the eggs or adults of *Z. arboreus* (Inoue & Tsuji, 2016; Kusano et al., 2005; Mori, 1992). Therefore, site‐specific predation risk differences, such as those between arboreal and terrestrial sites, should be further studied. In addition, focus should be given to the genetic differences between clades (Matsui et al., 2019), which might exhibit different behavioural features.

In conclusion, we provide novel insights into the evolution and adaptivity of foam nesting and oviposition site use in *Z. arboreus*, thereby making an important contribution to animal ecology.

## AUTHOR CONTRIBUTIONS

**Yukio Ichioka:** Data curation (equal); methodology (equal); writing – original draft (equal). **Hisashi Kajimura:** Methodology (equal); project administration (equal); supervision (equal).

## CONFLICT OF INTEREST STATEMENT

The authors declare that they have no competing interests.

## Data Availability

We deposited all the data obtained in this study in a repository, figshare (https://doi.org/10.6084/m9.figshare.23667387).
